# HbA_1c_ Variability as an Independent Risk Factor for Diabetic Retinopathy in Type 1 Diabetes: A German/Austrian Multicenter Analysis on 35,891 Patients

**DOI:** 10.1371/journal.pone.0091137

**Published:** 2014-03-07

**Authors:** Julia M. Hermann, Hans-Peter Hammes, Birgit Rami-Merhar, Joachim Rosenbauer, Morten Schütt, Erhard Siegel, Reinhard W. Holl, on behalf of the DPV Initiative the German BMBF Competence Network Diabetes Mellitus

**Affiliations:** 1 Institute of Epidemiology and Medical Biometry, ZIBMT, University of Ulm, Ulm, Germany; 2 5th Medical Department, Medical Faculty Mannheim, University of Heidelberg, Mannheim, Germany; 3 Department of Pediatrics and Adolescent Medicine, Medical University Vienna, Vienna, Austria; 4 Institute for Biometrics and Epidemiology, German Diabetes Centre, Leibniz Centre at Heinrich-Heine University Düsseldorf, Düsseldorf, Germany; 5 Department of Internal Medicine I, Medical University of Lübeck, Lübeck, Germany; 6 Department of Internal Medicine II, St. Josefs Hospital Heidelberg, Heidelberg, Germany; La Jolla Institute for Allergy and Immunology, United States of America

## Abstract

**Objective:**

This study aimed to analyze the effect of HbA_1c_ variability on the occurrence of diabetic retinopathy in type 1 diabetes patients.

**Patients and Methods:**

35,891 patients with childhood, adolescent or adult onset of type 1 diabetes from a large multicentre survey, the German/Austrian prospective documentation system (DPV), were analysed. Cox proportional hazard models were used to examine whether intra-individual HbA_1c_ variability expressed as variation coefficient is an independent risk factor for the occurrence of diabetic retinopathy.

**Results:**

Kaplan-Meier curves stratified by median HbA_1c_ and variation coefficient revealed that retinopathy-free survival probability is lower when both median HbA_1c_ and HbA_1c_ variability are above the 50^th^ percentile. Cox regression models confirmed this finding: After adjustment for age at diabetes onset, gender and median HbA_1c_, HbA_1c_ variability was independently associated with the occurrence of diabetic retinopathy. Time-covariate interactions used to model non-proportionality indicated an effect decreasing with duration of diabetes for both median HbA_1c_ and HbA_1c_ variability. Predictive accuracy increased significantly when adding HbA_1c_ variability to the Cox regression model.

**Conclusions:**

In patients with type 1 diabetes, HbA_1c_ variability adds to the risk of diabetic retinopathy independently of average metabolic control.

## Introduction

Diabetic retinopathy (DR) is the most frequent microvascular complication in patients with diabetes. It is well established that chronic hyperglycemia is one of the main risk factors for DR [1]. In addition, some recent analyses addressed the effect of HbA_1c_ variability on DR and related outcomes, as patients may show a wide variation in their long-term glycemic control, despite having similar average HbA_1c_ values [2]. Kilpatrick *et al.* [3] stated that longer-term glucose variability expressed as HbA_1c_ fluctuations contributed to the risk of DR in type 1 diabetes, whereas short-term glucose instability was no additional risk factor in the development of microvascular complications [4]. Hietala *et al*. [5] found HbA_1c_ variability to be associated with an increased risk of retinopathy requiring laser treatment in type 1 diabetes. Rodríguez-Segade *et al.* [6] reported that higher HbA_1c_ variability led to an increased risk of progression of nephropathy, independently of updated mean HbA_1c_. In contrast, Penno *et al.* [7] suggested that long-term fluctuation was no independent correlate of retinopathy in type 2 diabetes. Due to these inconsistent findings for different outcomes, further studies on the relationship between HbA_1c_ variability and DR are needed. Knowledge of whether highly varying HbA_1c_ values increase the risk of DR might help to improve diabetes management.

## Patients and Methods

### Ethics Statement

Analysis of anonymized routine data within the German/Austrian Diabetes Prospective Documentation Initiative (DPV) was approved by the Ethics Committee of the Medical Faculty of the University of Ulm.

### Patients

The DPV is a nationwide multicenter survey which by March 2013 comprised n = 83,856 patients with type 1 diabetes. Participating centers and data collection methods have been reported previously [8]. A total of 35,891 patients fulfilled the inclusion criteria which were as follows: availability of at least one retinal examination, and at least five HbA_1c_ values prior to the first occurrence of retinopathy or the last retinopathy examination. The latter exclusion criterion leads to more reliable estimates of the HbA_1c_ variability, as only patients with regular center attendance are included [9]. In addition, we repeated the analysis in patients with a minimum of four or six HbA_1c_ values. Assessment of diabetic retinopathy was performed according to the guidelines of the German Diabetes Association [10] and has been described before [11]. In brief, trained ophthalmologists used direct funduscopy in mydriasis to grade DR according to the modified Airlie House Classification/ETDRS standards [12]. The “multiple of the mean” transformation method was used to mathematically standardize HbA_1c_ values to the DCCT reference range (20.7–42.6 mmol/mol, 4.05–6.05%) in order to adjust for between-laboratory differences [13]. Further variables studied were duration of diabetes, gender and age at diagnosis in categories (<5 years, 5–<10 years, 10–<15 years, 15–<20 years and ≥20 years).

### Statistical Analysis

Statistical analysis was performed using SAS 9.3 (Statistical Analysis Software, SAS Institute Inc., Cary, NC, USA). Patient characteristics are presented as median with lower and upper quartile (median [Q1–Q3]) for continuous variables and as percentage for categorical variables. Differences between groups were analyzed by Mann-Whitney test. Average HbA_1c_ was calculated for each patient as the median of HbA_1c_ assessments during the individual observation time (HbA_1c_-MEDIAN). We determined a normalized measure of variability, the coefficient of variation (CV): Intra-individual standard deviation (SD) was divided by mean HbA_1c_ in order to correct for higher SDs due to larger absolute values (CV = SD/MEAN*100). Spearman’s rank correlation coefficient (*r_s_)* was computed to assess the strength of the association between median HbA_1c_ and HbA_1c_ variability. There was virtually no correlation between HbA_1c_-MEDIAN and CV (*r_s_* = −0.05, 95% CI −0.06, −0.04), whereas median HbA_1c_ and HbA_1c_-SD were weakly associated (*r_s_* = 0.27, 95% CI 0.25, 0.28). Hence, we used CV as variability measurement in order to avoid collinearity.

Kaplan-Meier curves describe the occurrence of retinopathy in relation to diabetes duration. Log-rank test was used for comparisons among strata. Patients who did not develop retinopathy during their individual observation time were right-censored. Multiple Cox regression models with duration of diabetes as time-scale were used to simultaneously consider the effect of independent variables. Model 1 included gender, age at diagnosis and median HbA_1c_ as covariates, Model 2 incorporated HbA_1c_-CV in addition. Proportionality assumption and functional form of covariates were checked by testing time-covariate interactions and by martingale residual plots. Non-proportionality was modeled by time-covariate interactions where necessary. Results are presented as hazard ratios (HR) and their corresponding 95% confidence intervals (CI). P<0.05 of a two-sided test was considered statistically significant. To compare the performance of the models, we calculated Gönen and Heller’s c-index [14] which is a concordance probability estimate that ranges between 0.5 and 1.0, with 1.0 representing perfect concordance between predicted and observed survival time. Being an extended version of the area under the receiver operating characteristic (ROC) curve that holds for censored data in the context of Cox regression models, it measures how well a model discriminates between different responses. Corresponding confidence intervals indicate whether c-indices differ significantly.

Examination of patients with at least four or at least six HbA_1c_ measurements led to similar results (data not shown).

## Results

Median age at the end of the individual observation time was 16.2 [13.1–18.0] years, and median diabetes duration was 6.4 [3.6–10.0] years. 52.3% of patients were male. Patients not included due to the lack of a retinal examination or less than five HbA_1c_ values documented were older (19.8 [13.4–45.4] years, p<0.0001) and had shorter duration of diabetes (5.6 [1.3–15.7] years, p<0.0001). However, since we investigate the additional effect of glycemic variability on the development of DR, rather than the prevalence of DR, we consider a potential selection bias to be irrelevant. 22.7% of the patients included were younger than 5 years at onset, 34.7% and 31.7% were 5-<10 years and 10-<15 years old, respectively. In 4.8% and 6.1% of the patients, age at onset was 15-<20 years and ≥20 years, respectively. Median number of HbA_1c_ values per patient during one year was 4.3 [3.5–5.3]. HbA_1c_-MEDIAN of participants was 59 [52–67] mmol/mol (7.5 [6.9–8.3] %), HbA_1c_-CV was 17.9 [12.7–25.1] %.

**Figure 1:**
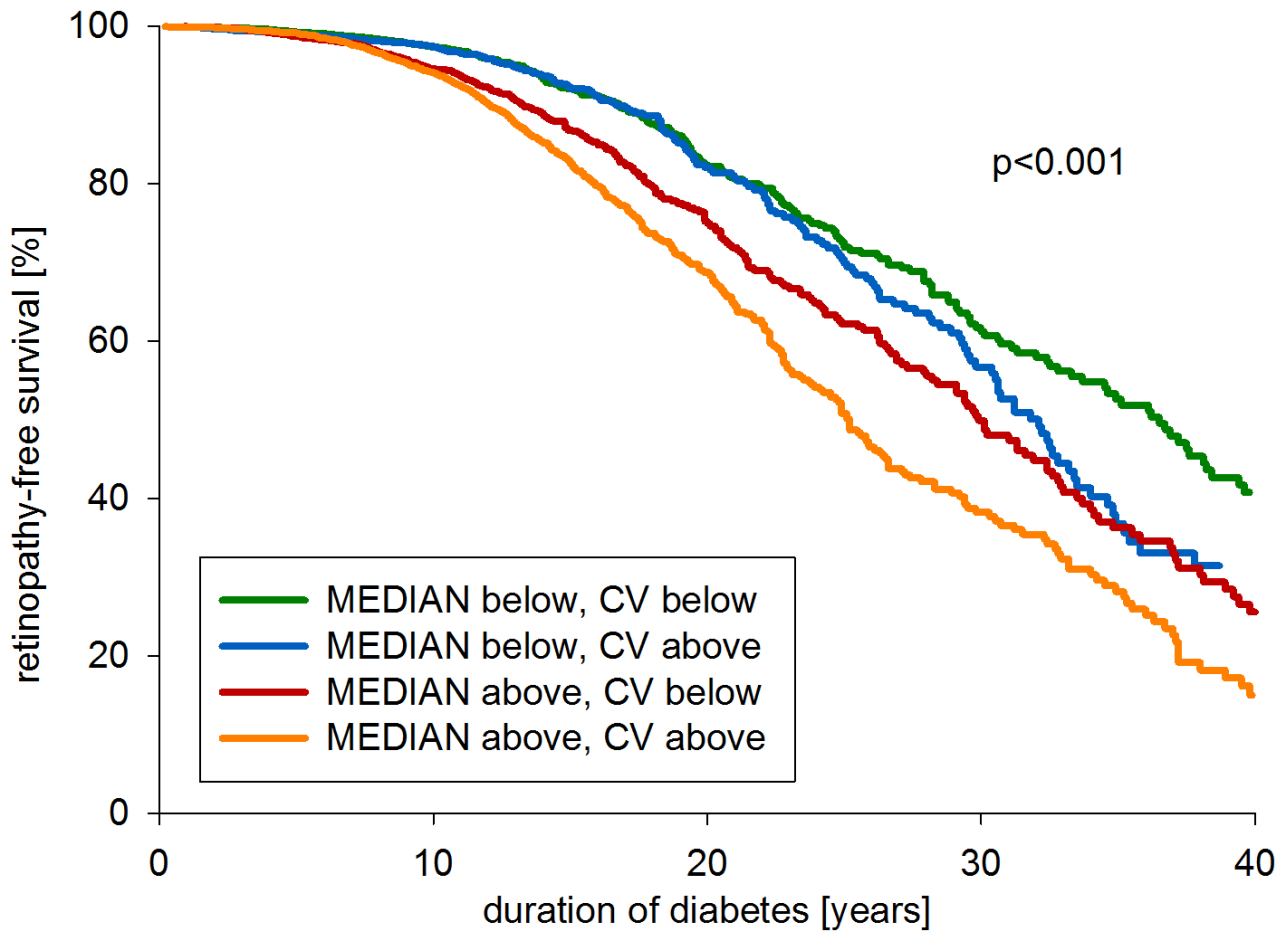
Kaplan Meier curves for retinopathy-free survival according to intrapersonal HbA_1c_-MEDIAN and HbA_1c_-CV above/below 50^th^ group percentile. Green line: HbA_1c_-MEDIAN below, HbA_1c_-CV below 50^th^ group percentile. Blue line: HbA_1c_-MEDIAN below, HbA_1c_-CV above 50^th^ group percentile. Red line: HbA_1c_-MEDIAN above, HbA_1c_-CV below 50^th^ group percentile. Orange line: HbA_1c_-MEDIAN above, HbA_1c_-CV above 50^th^ group percentile. Patients were assigned to strata based on group-specific 50^th^ percentiles according to duration of diabetes, age and gender. Log-rank test was used for comparisons among strata.

HbA_1c_ variability correlated negatively with duration of diabetes (*r_s_* = −0.34, 95% CI −0.35, −0.33, p<0.001). In order to account for this association, we assigned patients to groups according to duration of diabetes, age and gender and determined respective group-specific 50^th^ percentiles for HbA_1c_ and HbA_1c_-CV. We then assigned patients to groups with HbA_1c_-MEDIAN and HbA_1c_-CV above and below the respective 50^th^ group-specific percentiles and computed Kaplan-Meier curves (Fig. 1). Retinopathy-free survival was lowest (highest) when both median HbA_1c_ and HbA_1c_ variability were in the upper (lower) half (p<0.001).

In order to investigate the effect of age at onset, gender and HbA_1c_ simultaneously, we calculated multiple Cox regression models. We included first-order interaction terms between duration of diabetes and HbA_1c_-MEDIAN or HbA_1c_-CV to account for non-proportionality of these variables. All potential confounders except female gender were significantly related to retinopathy; age at onset <5 years was protective (Table 1, Model 1). Higher HbA_1c_-MEDIAN was associated with higher risk for retinopathy, but the effect decreased slightly with time (annual decrease in HR per one mmol/mol HbA_1c_-MEDIAN increase: 0.993; 95% CI 0.993, 0.994, p<0.001). At ten years of duration of diabetes, an increase of one mmol/mol HbA_1c_-MEDIAN was associated with a 3.1% higher risk of DR. HbA_1c_ variability led to an additional rise in risk (3.5% higher risk of DR per one unit increase of HbA_1c_-CV at ten years of duration of diabetes) (Model 2). Discriminative ability of the Cox regression model measured by Gönen and Heller’s c-index increased significantly from 0.831 (95% CI 0.825, 0.837) to 0.868 (95% CI 0.863, 0.873) after adding HbA_1c_ variability to Model 1.

**Table 1:** Relative risk (HR) estimated from multiple Cox regression for the association between HbA_1c_ and development of diabetic retinopathy, adjusted for demographic variables.

	Model 1		Model 2	
Variables	HR [95% CI]	P	HR [95% CI]	P
Female gender	0.984 [0.896–1.080]	0.734	0.974 [0.887–1.069]	0.573
Age at onset <5 years	1.0		1.0	
Age at onset 5–<10 years	1.577 [1.359–1.830]	<0.001	1.512 [1.301–1.757]	<0.001
Age at onset 10–<15 years	1.907 [1.606–2.263]	<0.001	1.642 [1.379–1.956]	<0.001
Age at onset 15–<20 years	1.607 [1.249–2.068]	<0.001	1.242 [0.958–1.610]	0.103
Age at onset ≥20 years	2.370 [2.020–2.782]	<0.001	2.238 [1.902–2.634]	<0.001
HbA_1c_-MEDIAN (mmol/mol)	1.106 [1.102–1.110]	<0.001	1.098 [1.094–1.102]	<0.001
HbA_1c_-MEDIAN * diabetes duration	0.993 [0.993–0.994]	<0.001	0.994 [0.993–0.994]	<0.001
HbA_1c_-CV (%)	–		1.110 [1.100–1.121]	<0.001
HbA_1c_-CV * diabetes duration	–		0.993 [0.992–0.994]	<0.001
c-index [95% CI]	0.831 [0.826–0.837]		0.868 [0.863–0.873]	

Results are presented as hazard ratios and their corresponding 95% confidence intervals. Time scale: duration of diabetes in years.

Example: HR for HbA_1c_-MEDIAN for ten years of duration of diabetes: HR = 1.106*0.993^10^ = 1.031.

## Discussion

Our study in patients with type 1 diabetes demonstrated that HbA_1c_ variability is an independent risk factor for diabetic retinopathy. In a multiple Cox regression model, HbA_1c_ variability was significantly associated with DR, independent of median HbA_1c_ value. For both median HbA_1c_ and HbA_1c_-CV, the contribution was lower with longer duration of diabetes. This finding may be explained by genetic susceptibility: Some patients with poor glycemic control do not develop DR even over long time periods [15]. Discriminative ability of the Cox regression model improved significantly compared to a model not containing any fluctuation measurement. Concordance between predicted and observed survival time was good, although we only included gender, age at onset and glycemic control as predictor variables. Adding variables like hypertension, dyslipidemia or ethnicity could improve the overall prediction, but not all of these variables were clearly shown to have an important effect on DR [11], [16]. Furthermore, since our investigation focused on the additional impact of variability, we chose a Cox regression model including demographic variables and metabolic control only.

Our database is large and differences in c-index are small, but significant; therefore, the issue of statistical significance versus clinical relevance has to be addressed. Considering the fact that the pathogenesis of DR is complex, a greater improvement in predictive accuracy as a result of adding one variable only is not to be expected. In addition, point estimates expressed by hazard ratios and their associated confidence intervals revealed clear effects of HbA_1c_ variability on the risk of DR.

Kilpatrick (2012) [17] mentioned several possible reasons as to why HbA_1c_ variability might contribute to the risk of DR. He supposed that periods of hyperglycemia are ‘remembered’ and therefore the effect of HbA_1c_ variability could be caused by the same mechanism underlying the ‘metabolic memory’ phenomenon. Another explanation comprised the short-term ‘early worsening’. There could be insufficient time for long-term benefits in patients with fluctuating glycemic control. The author also suspected that patients with highly varying HbA_1c_ are those with suboptimal diabetes management.

The main strength of our study is the large number of patients and the long observation time. Possible limitations are the varying number of measurements per individual and various time intervals between two examinations. Moreover, data are collected at numerous diabetes centers with different rates of eye examination.

In conclusion, this large routine survey reveals that HbA_1c_ variability adds to the risk of diabetic retinopathy independently of average metabolic control. Our results and the possible explanations mentioned above allow the conclusion that continuous care results in better outcome compared to short interventions triggered by elevated HbA_1c_ values.
